# Multispectral PPG Sensing Framework for Noninvasive Glucose Prediction via Multi-Feature Fusion and Micro-Environmental Compensation

**DOI:** 10.3390/bioengineering13101231

**Published:** 2026-10-21

**Authors:** Lin Huang, Jie Wang, Yuanshuang Fan, Jiansen Lan, Haijun Luo, Rui Wang

**Affiliations:** 1 College of Electronic and Information Engineering, Chongqing Normal University, Chongqing 401331, China; 2024210511038@stu.cqnu.edu.cn (L.H.); 2023210511048@stu.cqnu.edu.cn (J.W.); 2024210511042@stu.cqnu.edu.cn (J.L.);; 2 State Key Laboratory of Ultrasound in Medicine and Engineering, College of Biomedical Engineering, Chongqing Medical University, Chongqing 400016, China; wangrui_wise@cqmu.edu.cn

**Keywords:** noninvasive glucose prediction, multispectral photoplethysmography, near-infrared spectroscopy, temperature and humidity compensation, XGBoost, interpretable machine learning

## Abstract

Noninvasive blood glucose prediction using near-infrared spectroscopy remains challenging due to weak glucose-specific absorption, tissue variability, and environmental interference. This study proposes a multispectral photoplethysmography (PPG)-based framework integrating multi-feature fusion, local temperature and humidity compensation, and machine learning regression. The system employs four near-infrared wavelengths (940, 1300, 1550, and 1650 nm) while simultaneously measuring finger contact-site temperature (T) and local relative humidity (H) within the finger clip. A 39-dimensional feature set incorporating optical ratios, pulse-wave morphology, frequency-domain descriptors, participant characteristics, and environmental parameters was constructed. Among six optimized regression models, Extreme Gradient Boosting (XGBoost) achieved the best performance in group-isolated 10-fold cross-validation, with R^2^ = 0.9135, root mean square error (RMSE) = 0.2991, and mean absolute relative difference (MARD) = 2.71%. Clarke error grid analysis (CEGA) showed that 97% of predictions fell within zone A and 100% within zones A and B. Feature ablation demonstrated that local temperature and humidity measurements improved prediction accuracy, with humidity providing a greater improvement than temperature. SHapley Additive exPlanations (SHAP) analysis quantified the contributions of optical, environmental, and participant-level features. A separate session-holdout evaluation yielded R^2^ = 0.7261 and MARD = 6.07%. These findings highlight the potential of the proposed framework for noninvasive glucose prediction. However, the small, predominantly healthy cohort and limited glucose range constrain generalizability, necessitating validation in larger, more diverse populations to establish performance in previously unseen participants and assess clinical applicability.

## 1. Introduction

Diabetes mellitus (DM), a leading contributor to the global burden of noncommunicable diseases, is associated with oxidative stress, the accumulation of advanced glycation end products (AGEs), and chronic inflammation—key mechanisms contributing to complications such as retinopathy, nephropathy, and cardiovascular disease [1,2]. According to the International Diabetes Federation (IDF), the global adult population living with diabetes has exceeded 589 million and is projected to reach 853 million by 2050, highlighting the substantial global burden of the disease [3]. This growing burden underscores the need for accurate and accessible glucose monitoring technologies. However, the pain and inconvenience associated with conventional invasive glucose testing can limit adherence to frequent monitoring, making noninvasive, sensitive, and low-power monitoring systems an important research direction in biomedical engineering.

Currently, several promising approaches to noninvasive glucose monitoring have emerged. These include interstitial fluid (ISF) extraction via microneedles [4,5,6], microwave and radiofrequency (RF) dielectric sensing [7,8,9,10], terahertz spectroscopy, and various optical detection methods such as near-infrared (NIR, 700–2500 nm) [11,12,13], mid-infrared (MIR) [14,15], Raman [16,17,18], and photoacoustic spectroscopy (PAS) [19]. Among these, NIR spectroscopy has attracted considerable attention because glucose exhibits overtone and combination absorption bands associated with molecular vibrations, including C–H and O–H bonds. However, in vivo spectroscopic measurements are strongly affected by tissue scattering, physiological variability, and motion artifacts. To capture optical variations associated with pulsatile blood volume changes, photoplethysmography (PPG) provides a complementary approach by monitoring microvascular blood volume fluctuations. PPG is widely used to monitor physiological parameters such as oxygen saturation [20,21], heart rate [22], respiration rate [23], and blood pressure [24]. Recent studies have also investigated its potential for noninvasive glucose estimation by exploiting glucose-related optical variations in multispectral PPG signals.

Recent advances in noninvasive blood glucose monitoring (NIBGM) have increasingly explored the integration of multispectral PPG with machine learning and deep learning techniques. However, existing approaches face two important challenges. First, studies employing relatively limited wavelength combinations (e.g., 660/940 nm [25], 940/1300 nm [26], or 530/1550 nm [27] may provide insufficient spectral information to distinguish weak glucose-related optical signals from interference caused by water absorption and variations in tissue properties. Second, although advanced learning architectures, such as multimodal long short-term memory networks (LSTMs) [28], cross-fusion Transformers [29], and deep neural networks [26,30], can improve feature representation and prediction performance, their limited interpretability may complicate the identification of physiologically meaningful predictors. Moreover, variations in temperature, humidity, and individual physiological characteristics can influence optical measurements at the sensor–skin interface, highlighting the need to account for these factors in model development.

**Figure 1:**
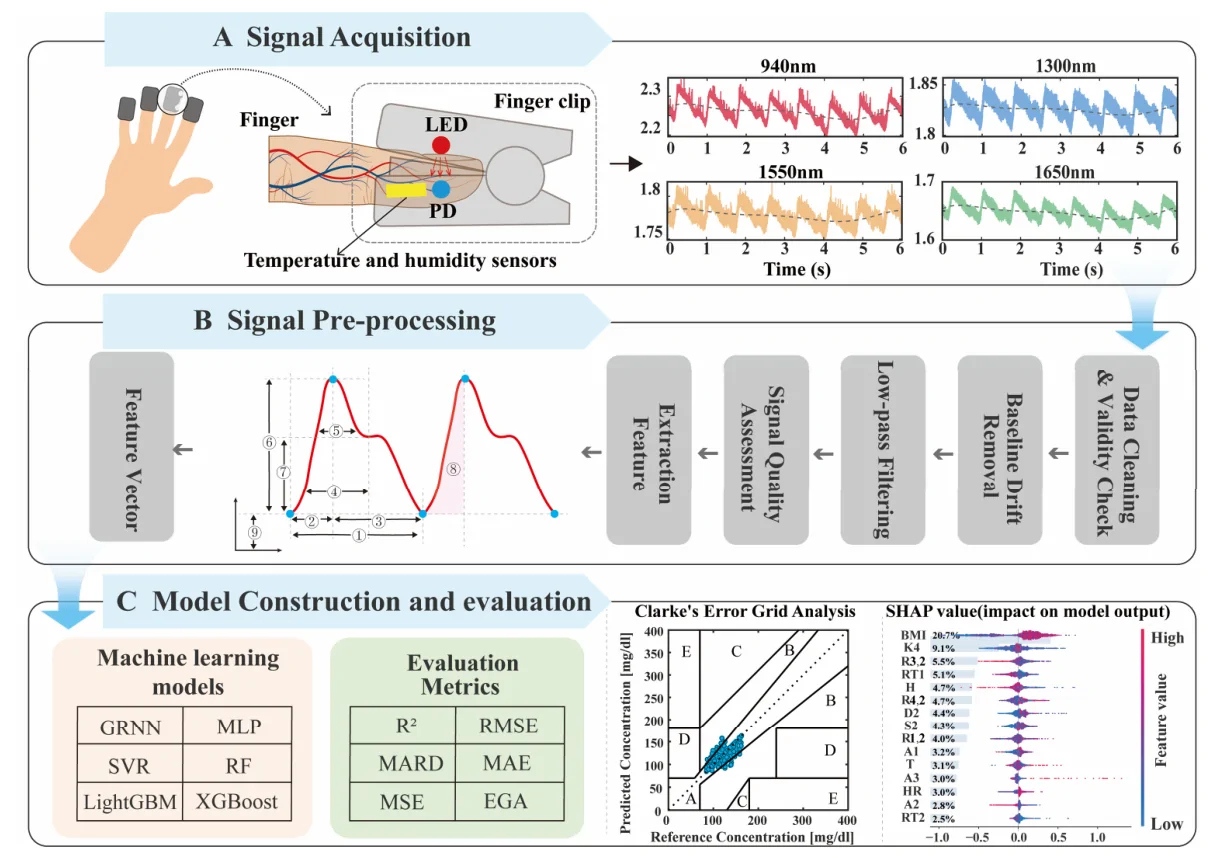
Overview of the proposed noninvasive glucose estimation framework. Panels (**A**, **B**, **C**) represent signal acquisition, signal preprocessing, and model construction and evaluation modules, respectively. Circled numbers ①–⑧ in panel (**B**) denote different morphological parameters extracted from the PPG waveform.

To address these measurement and modeling challenges, this study developed a noninvasive glucose prediction framework based on a four-wavelength near-infrared photoplethysmography (PPG) system operating at 940, 1300, 1550, and 1650 nm (Figure 1). The framework integrates multispectral signal acquisition (Figure 1A), signal preprocessing and multi-feature extraction (Figure 1B), and machine learning regression and evaluation (Figure 1C). Features derived from the PPG signals were combined with participant characteristics, temperature measured at the finger–sensor contact site, and the relative humidity of the adjacent air within the finger clip. Six regression models were compared to assess predictive performance, and a separate feature ablation analysis was conducted to evaluate the contributions of temperature and humidity to PPG-based prediction. SHapley Additive exPlanations (SHAP) analysis was applied to the full-feature model to examine the contributions of optical, environmental, and participant-level variables to model predictions. Finally, a session-holdout test was performed to evaluate predictive performance on held-out acquisition sessions. Together, these analyses provide a comprehensive evaluation of the proposed multi-feature fusion framework in terms of predictive accuracy, environmental feature contributions, model interpretability, and cross-session stability.

## 2. Materials and Methods

### 2.1. Principle of PPG-Based Glucose Measurement

**Figure 2:**
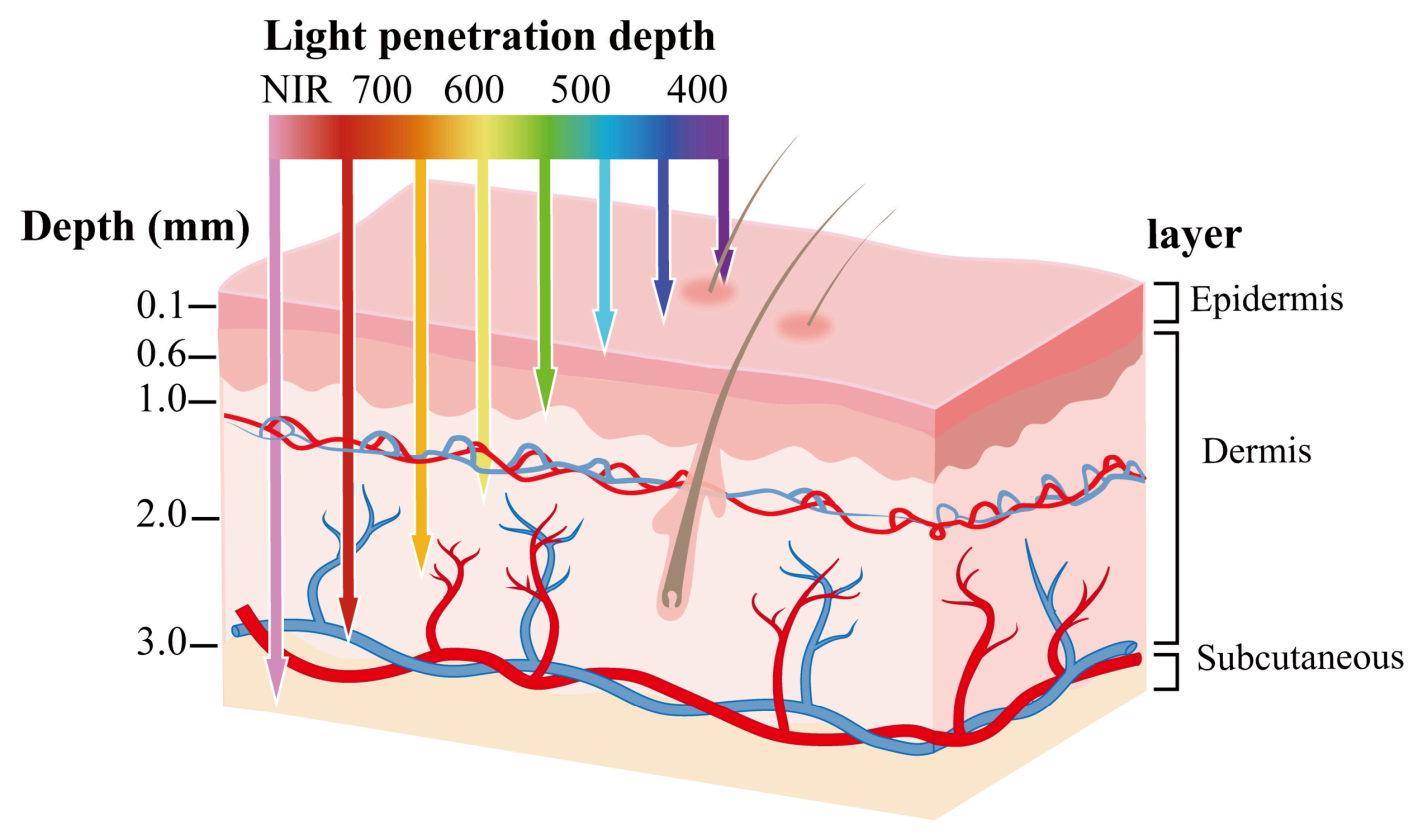
Light penetration depth at different wavelengths.

**Figure 3:**
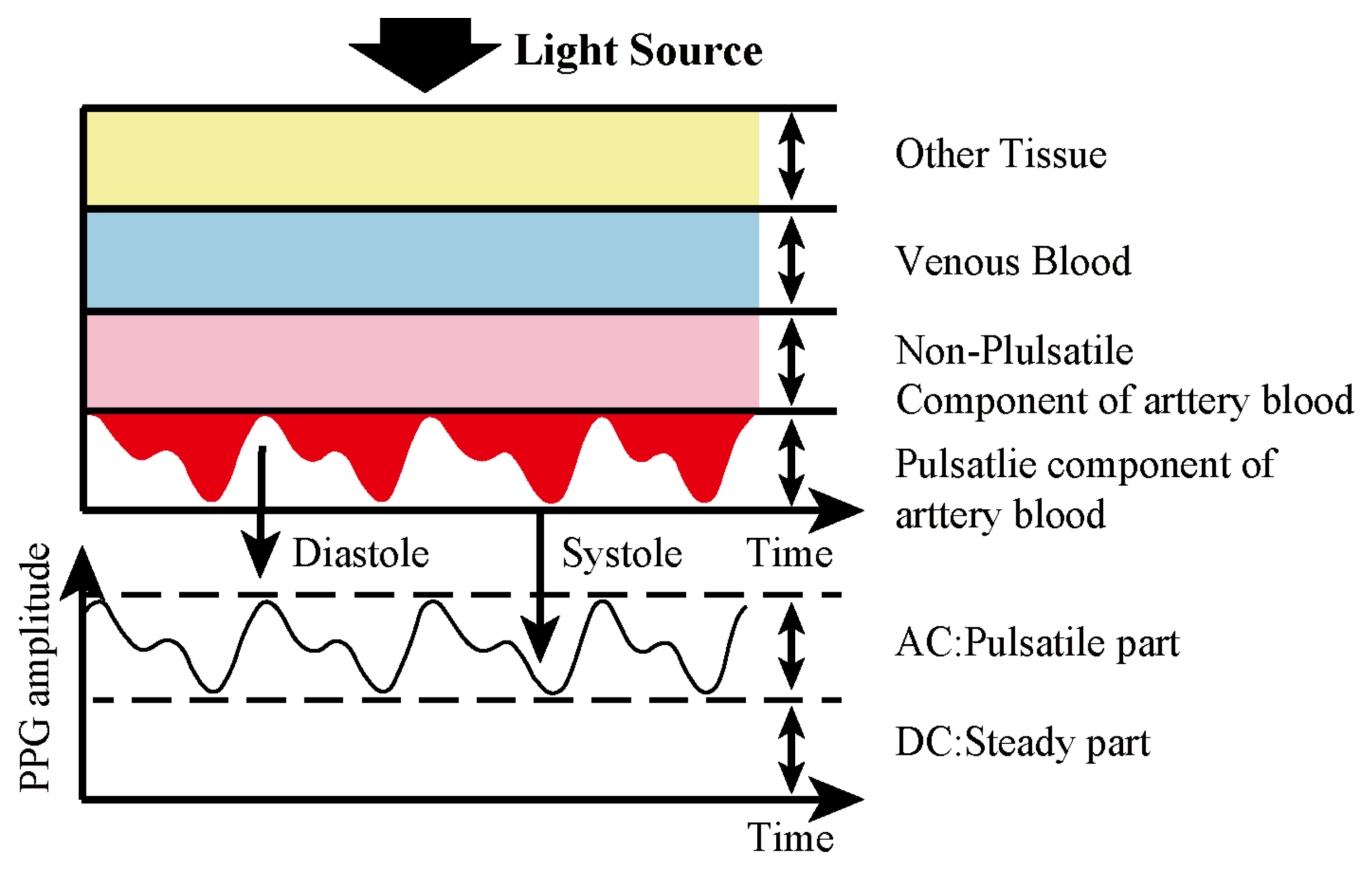
Composition and physiological origin of a typical PPG signal.

Optical penetration depth in human tissue is wavelength-dependent (Figure 2). Near-infrared (NIR, 750–2500 nm) light reaches the vascularized dermis and subcutaneous layers, providing high sensitivity to glucose-induced optical variations [31]. PPG captures these dynamics via pulsatile arterial absorption (AC) superimposed on static tissue attenuation (DC) (Figure 3) [32]. Fluctuations in blood glucose alter tissue refractive indices and modulate specific NIR absorption bands (notably overtone/combination C–H, O–H, and C–O resonances near 940, 1300, and 1550 nm) [33]. Governing this light–tissue interaction, the modified Beer–Lambert law models optical absorbance (A) as:

(1) $$A(\lambda )=\mathrm{lg}\left(\frac{I_{0}(\lambda )}{I(\lambda )}\right)=\varepsilon (\lambda )\cdot c\cdot d$$

where $I_{0}$ and I denote incident and transmitted light intensities, ε(λ) is the molar absorption coefficient, c represents glucose concentration, and d signifies optical path length [34]. Because these molecular perturbations induce subtle, non-linear variations in both PPG amplitude and wave morphology, absolute intensity regression remains vulnerable to physiological noise. Consequently, this framework extracts cross-wavelength ratio features across distinct spectral channels to eliminate inter-subject optical variations (e.g., skin thickness, melanin) and instrument drift [35], leveraging machine learning regression to achieve robust, highly specific glucose estimation.

### 2.2. Multispectral Hardware Platform and Environmental Sensing Architecture

**Figure 4:**
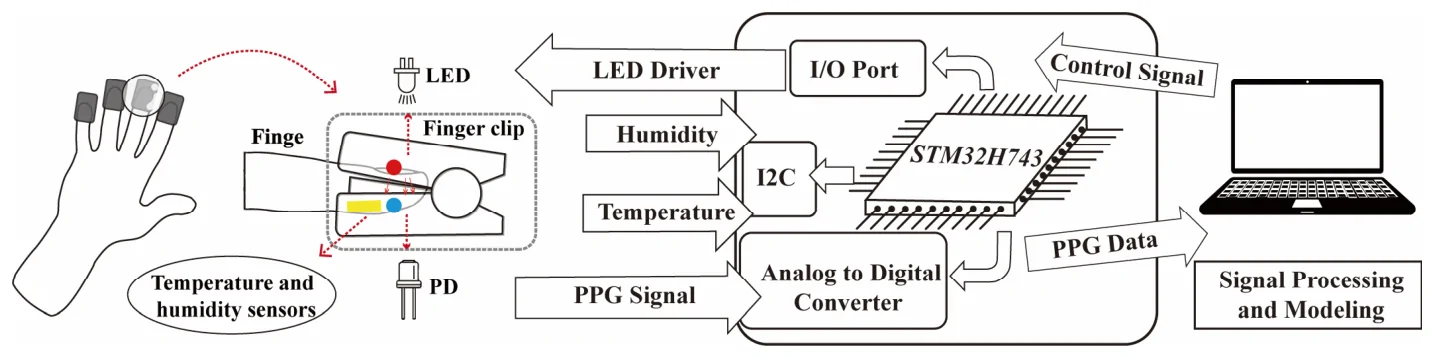
Hardware architecture of the multispectral PPG-based glucose sensing system.

**Figure 5:**
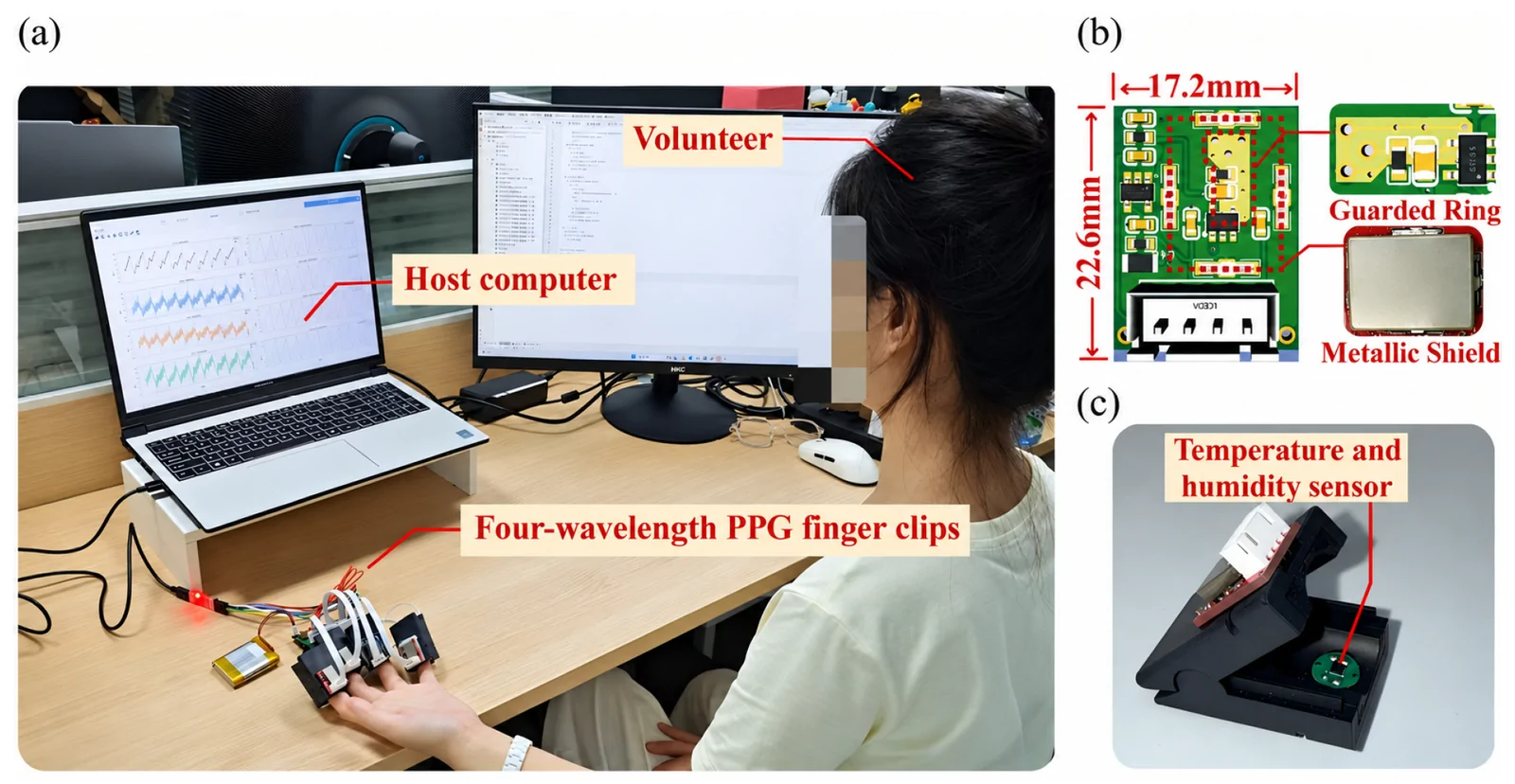
Experimental setup and signal acquisition system. (**a**) Four-channel fingertip PPG acquisition; (**b**) PCB layout design of the detection board; (**c**) finger-clip sensor module for the 1300 nm channel.

The multispectral PPG sensing system integrates custom-designed optoelectronic hardware with an embedded data acquisition system (Figure 4 and Figure 5). To maintain stable optical coupling and minimize motion artifacts, the acquisition module incorporates four ergonomic, spring-loaded finger clips lined with medical-grade silicone (Figure 5a). The illumination unit consists of four NIR LEDs centered at 940, 1300, 1550, and 1650 nm, driven by a multichannel constant-current circuit to capture glucose-related optical information across different wavelengths. Synchronous optical detection is performed at 1 kHz using a broadband InGaAs photodiode (LSIPD-L1, Beijing Lightsensing Technologies Ltd., Beijing, China, 800–1700 nm). To reduce electrical interference, the detection PCB incorporates a guard ring and a metallic shield (Figure 5b). An AHT30 digital temperature and humidity sensor (Aosong Electronics, Guangzhou, China) is embedded within the 1300 nm finger clip and positioned in direct contact with the finger surface. It measures the local temperature at the contact site and the relative humidity of the adjacent microenvironment at 1 Hz (Figure 5c).

Signal conditioning is managed via a low-noise transimpedance amplifier (TIA) and an active analog low-pass filter. A 32-bit microcontroller (STM32H743, STMicroelectronics, Geneva, Switzerland, ARM Cortex-M7) serves as the core processor, utilizing a 16-bit ADC paired with a Direct Memory Access (DMA) pipeline for zero-latency, synchronized sampling of four-channel PPG waveforms and environmental parameters. The digitized data streams are streamed to a host computer via USB for downstream feature extraction and machine learning modeling (Figure 4).

### 2.3. Subjects and Experimental Protocol

A total of 10 volunteers (9 healthy subjects and 1 individual with prediabetes) with Fitzpatrick skin phototypes II–IV were recruited for this study. The demographic characteristics are summarized in Table 1. All participants were fully informed of the study procedures and provided written informed consent prior to data collection. Ethical approval for all self-built datasets used in this study was obtained from the Ethics Committee of Chongqing Medical University (Approval No. 2026-138), and all procedures were conducted in accordance with the Declaration of Helsinki and applicable regulatory requirements.

To ensure hemodynamic stability, subjects rested in a seated position for at least 5 min before each measurement session (Figure 5a). The fingertip was cleaned with an alcohol swab and allowed to air dry to minimize optical interference from surface contaminants. For each measurement session, four-wavelength PPG signals were synchronously recorded for 1 min per trial. This procedure was repeated three times consecutively at the same fingertip location, with the finger briefly removed and reinserted between trials to simulate independent re-positioning. All three repeated trials corresponding to the same reference glucose value were treated as a single “acquisition group” to account for the correlation structure introduced by repeated measurements. Each subject was evaluated under seven metabolic conditions: fasting, and 1 h and 2 h postprandial after breakfast, lunch, and dinner. Reference blood glucose was measured immediately after each PPG acquisition session using an FDA-approved glucometer (Accu-Chek Performa, Roche Diagnostics, Mannheim, Germany) to ensure strict temporal alignment.

A total of 682 valid samples were collected across all subjects and conditions. To prevent data leakage caused by repeated measurements from the same subject and same metabolic state, the dataset was partitioned at the acquisition-group level, not at the individual sample level. Specifically, all three repeated trials within each acquisition group were assigned exclusively to either the training set or the test set as a single unit. A subject-stratified random sampling strategy was adopted: approximately 20% of the acquisition groups from each subject were randomly selected as the test set, and the remaining 80% were used as the training set. This strategy ensures that (1) samples within the same acquisition group are not split, and (2) every subject contributes data to both training and test sets, avoiding evaluation bias caused by the complete absence of any particular individual from the test set.

### 2.4. Signal Processing and Feature Fusion

**Figure 6:**
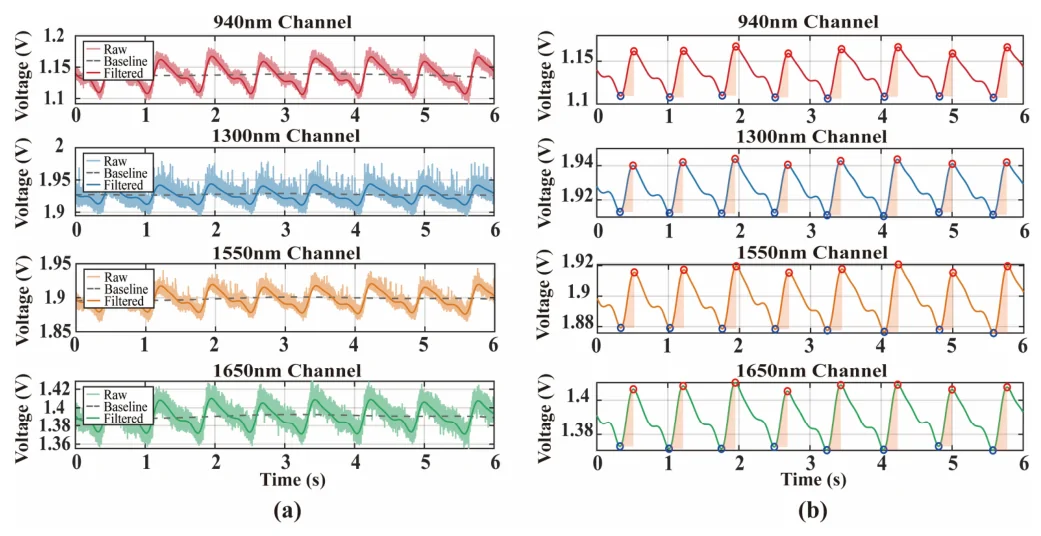
Schematic illustration of multi-wavelength PPG signal preprocessing and feature extraction. (**a**) Comparison of raw signals, baseline drift, and filtered signals across four wavelength channels; (**b**) feature extraction results for the four wavelength channels.

Raw PPG waveforms are susceptible to respiratory activity, finger motion, contact pressure variations, and optical path disturbances, typically manifesting as low-frequency baseline drift superimposed with high-frequency noise. To enhance signal quality and temporal consistency, a systematic preprocessing and feature extraction pipeline was established (Figure 6), including detrending, filtering, outlier removal, feature computation, and statistical aggregation.

For detrending, a symmetric sliding-window polynomial modeling approach was employed. A sixth-order polynomial was fitted within a 6 s window with a 3 s step size, yielding a 50% overlap between adjacent windows. This overlap setting was chosen to ensure smooth baseline continuity while maintaining computational efficiency. An overlap-and-add (OLA) strategy was then applied to fuse baseline estimates across windows, yielding a globally smooth and continuous detrended signal. Subsequently, a third-order Butterworth low-pass filter with a cutoff frequency of 4.5 Hz was applied using zero-phase filtering to preserve the dominant cardiac components while effectively suppressing high-frequency noise. The third-order configuration was selected as it offers a reasonable trade-off between roll-off sharpness and passband ripple, adequate for preserving PPG morphological features without introducing phase distortion.

Signal quality control employed a baseline variation-based anomaly metric with adaptive thresholding based on the median absolute deviation (MAD), defined as the median of the absolute deviations from the median. This procedure was used to remove abnormal and nonstationary segments. A cross-wavelength consistency constraint was further applied, retaining only segments that satisfied the quality criteria across all wavelength channels. Finally, a continuous 30 s high-quality, temporally aligned segment was selected for feature extraction and modeling.

For feature extraction, considering that the absolute amplitude of PPG signals is strongly affected by inter-individual variations, such as skin optical properties, hemodynamic conditions, and finger geometry, individual amplitude-based features may exhibit limited robustness. To mitigate these effects, this study extracted 36 statistical features across time, frequency, and morphological domains, as illustrated in Figure 6. The preprocessing pipeline includes baseline correction and filtering of the raw signals from the four wavelength channels, followed by the extraction of multidimensional features from the processed signals.

To comprehensively characterize the optical dynamic variations associated with blood glucose, the extracted features include: cross-wavelength amplitude ratios ($R_{k,2}$), perfusion indices ($PI_{k}$), skewness ($\gamma_{k}$), kurtosis ($K_{k}$), peak means ($S_{k}$), and valley means ($D_{k}$). Frequency-domain and temporal parameters are also incorporated, such as the maximum magnitude of the primary frequency ($A_{k}^{max}$), rise time ($RT_{k}$), and the average area under the rising segment ($A_{k}$). Additionally, heart rate (HR) and local conditions at the finger–sensor interface, including contact-site temperature (T) and the relative humidity of the adjacent air within the finger clip (H), are included to ensure environmental stability. All features are calculated based on complete cardiac cycles and jointly characterized across multiple wavelength channels. To enhance the model’s adaptability across different individuals, static physiological traits such as age, gender, and Body Mass Index (BMI) are integrated into the input variables. Ultimately, each sample is represented by a 39-dimensional multimodal feature vector:

(2) $$F_{\mathrm{input}}=\left[R_{k,2},PI_{k},\gamma_{k},K_{k},\ldots ,\mathrm{BMI}\right]$$

In the equation, $F_{\mathrm{input}}$ represents the input feature vector of the model, which is constructed by concatenating multi-dimensional heterogeneous features according to their information types. The variable k denotes the channel index, where k = 1, 2, 3, 4 correspond to the NIR light source channels with wavelengths of 940 nm, 1300 nm, 1550 nm, and 1650 nm, respectively.

At the feature level, an additional quality control mechanism was introduced to further eliminate abnormal features induced by low-quality signals. Specifically, samples affected by amplitude saturation, transient spike interference, irregular cardiac cycles, or abnormal spectral energy distributions were excluded, thereby reducing the impact of corrupted signals on the feature distribution.

### 2.5. Machine Learning Framework

In this study, six representative machine learning regression models were evaluated for glucose prediction, including the Generalized Regression Neural Network (GRNN), Support Vector Regression (SVR), Random Forest (RF), Light Gradient Boosting Machine (LightGBM), Extreme Gradient Boosting (XGBoost), and Backpropagation Neural Network (BP). These models represent different learning paradigms, including kernel-based statistical learning (SVR), tree-based ensemble methods (RF, LightGBM, XGBoost), probabilistic radial basis function networks (GRNN), and feedforward neural networks trained by backpropagation (BP), providing a diverse regression modeling framework. To optimize the hyperparameters of each model, automated hyperparameter optimization was performed using the Optuna framework [36] with a Tree-structured Parzen Estimator (TPE) sampling strategy for Bayesian optimization. The optimization objective was to minimize the mean root mean square error (RMSE) across 10-fold cross-validation (CV). Model performance was evaluated using multiple statistical metrics, including the coefficient of determination (R^2^), mean absolute error (MAE), mean squared error (MSE), RMSE, and mean absolute relative deviation (MARD), to assess prediction accuracy comprehensively.

Additionally, Clarke error grid analysis (CEGA) [37] was employed to assess the clinical implications of glucose prediction errors. Zone A represents clinically accurate predictions, whereas zone B includes deviations that are unlikely to lead to inappropriate treatment decisions. Predictions in zones C–E may lead to unnecessary treatment, failure to detect conditions requiring intervention, or treatment in the wrong direction. The percentages of predictions in zones A and B were therefore used to summarize clinical acceptability within the evaluated dataset.

## 3. Results

### 3.1. Model Performance and Clinical Accuracy

The predictive capabilities of six optimized machine learning regression models—GRNN, SVR, RF, LightGBM, XGBoost, and BPNN—were benchmarked using 10-fold acquisition-group-wise cross-validation (Table 2). All three repeated trials within each acquisition group were assigned to the same fold. Consequently, no acquisition group was shared between the training and validation subsets in any fold. Because acquisition groups from the same participant could be assigned to different folds, the resulting performance represents generalization across acquisition groups rather than validation on previously unseen participants.

Overall, the gradient-boosting models, XGBoost and LightGBM, exhibited the best performance. XGBoost achieved the highest coefficient of determination (R^2^ = 0.9135) and the lowest root mean square error (RMSE = 0.2991), mean absolute error (MAE = 0.1740), mean square error (MSE = 0.0900), and mean absolute relative deviation (MARD = 2.71%). LightGBM showed comparable performance (R^2^ = 0.9122, MARD = 2.96%), whereas GRNN (R^2^ = 0.8695, MARD = 3.85%) and SVR (R^2^ = 0.8710, MARD = 3.71%) showed lower predictive performance.

**Figure 7:**
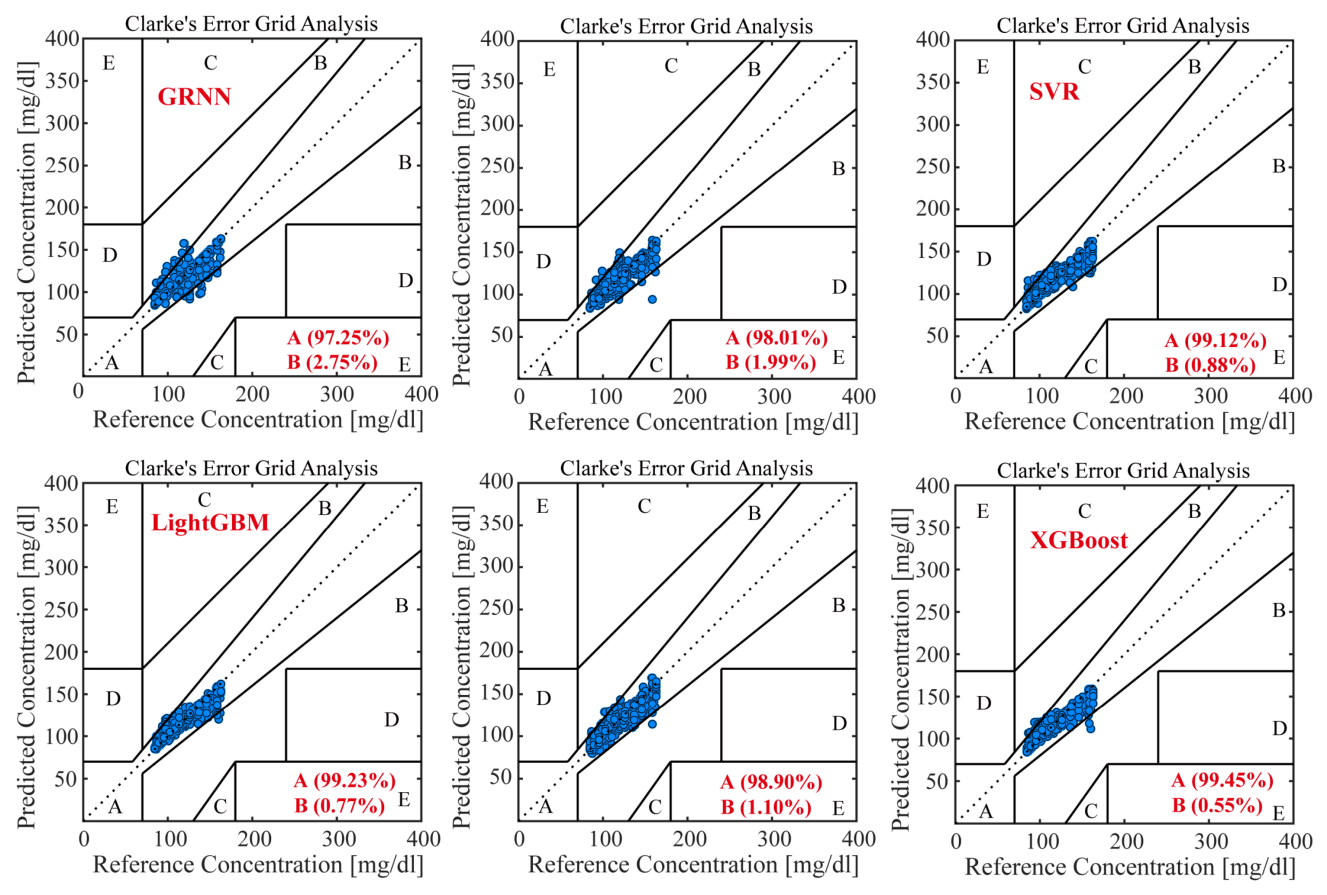
CEGA comparing six regression models for clinical safety evaluation.

To evaluate clinical safety, CEGA was conducted across all six models (Figure 7). The XGBoost model achieved exemplary clinical accuracy, with 99.45% of predicted blood glucose values residing within Zone A (clinically accurate) and 100% contained within the combined clinically acceptable Zones A and B (0% in Zones C, D, and E). In comparison, while SVR (99.12% in Zone A) and LightGBM (99.23% in Zone A) also exhibited high clinical safety, their proportion of predictions in Zone B was noticeably higher. These results confirm that the XGBoost framework provides both optimal statistical precision and uncompromising clinical reliability for non-invasive glucose estimation.

### 3.2. Temperature and Humidity Ablation Analysis

**Figure 8:**
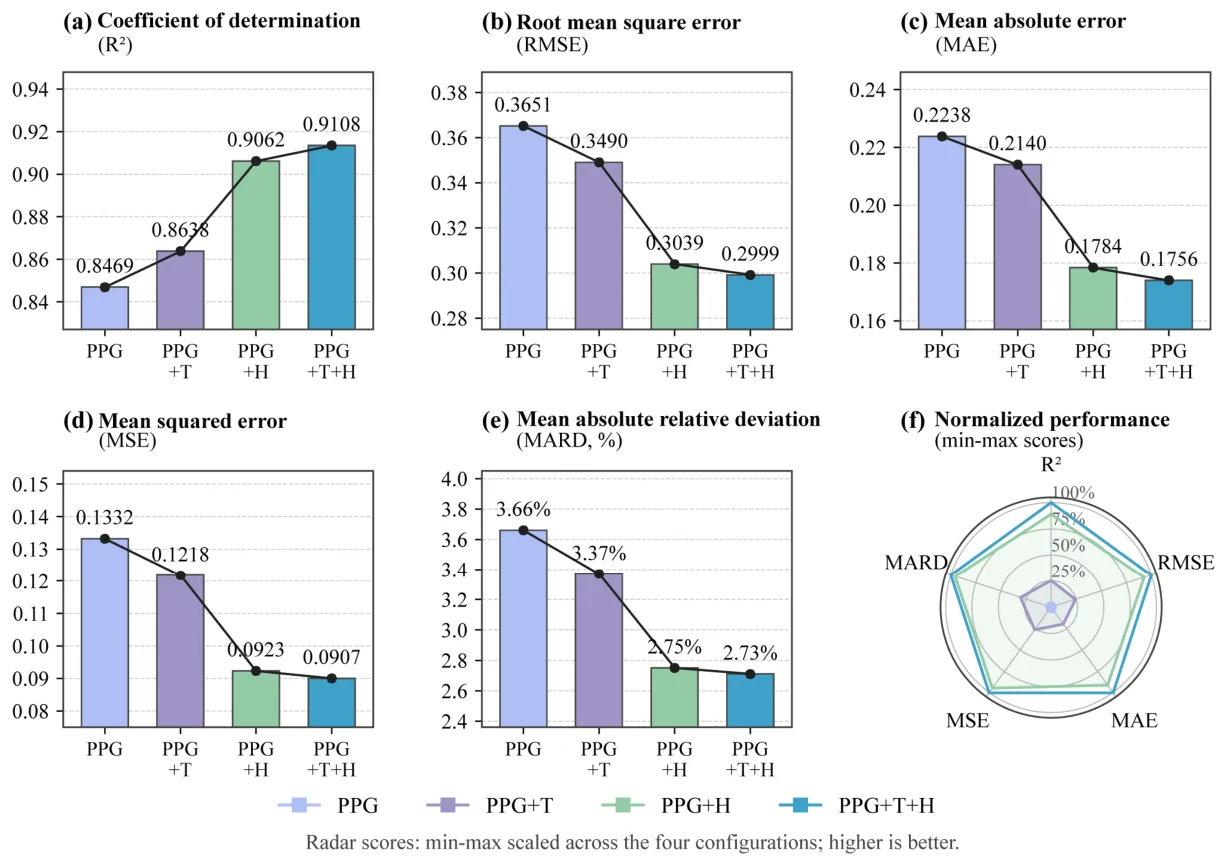
Effects of temperature and humidity features on model performance in an ablation analysis comparing four input configurations: PPG alone, PPG with temperature (PPG + T), PPG with humidity (PPG + H), and PPG with both temperature and humidity (PPG + T + H). (**a**) Coefficient of determination (R^2^); (**b**) root mean square error (RMSE); (**c**) mean absolute error (MAE); (**d**) mean squared error (MSE); (**e**) mean absolute relative difference (MARD); and (**f**) radar chart summarizing the five performance metrics. T and H denote temperature and humidity, respectively.

To evaluate the individual and combined contributions of temperature (T) and humidity (H) to blood glucose prediction, a feature ablation analysis was conducted using XGBoost with four input configurations: PPG-only, PPG + T, PPG + H, and PPG + T + H (Figure 8a–f). T denotes the temperature measured at the finger–sensor contact site, and H denotes the relative humidity of the adjacent air within the finger clip. All four configurations used features extracted from the multispectral PPG signals; age, sex, BMI, and other subject-level characteristics were excluded. This analysis was separate from the SHAP analysis of the full 39-dimensional model.

The PPG-only configuration achieved an R^2^ of 0.8469 and a MARD of 3.66%. Adding T increased R^2^ to 0.8638 and reduced MARD to 3.37%, whereas adding H increased R^2^ to 0.9062 and reduced MARD to 2.75% (Figure 8a,e). Relative to PPG-only, the reductions in MARD were 0.29 percentage points with T and 0.91 percentage points with H. The combined PPG + T + H configuration achieved the best observed performance, with R^2^ = 0.9108, RMSE = 0.2999, MAE = 0.1756, MSE = 0.0907, and MARD = 2.73% (Figure 8a–e). Compared with PPG + T, adding H further reduced MARD by 0.64 percentage points; compared with PPG + H, adding T reduced it by 0.02 percentage points. Thus, across the evaluated configurations, H was associated with the larger incremental improvement, while the additional gain from T after H had been included was small.

### 3.3. SHAP-Based Feature Importance Profile

To quantify feature attribution and evaluate the relative importance of individual input variables within the optimized XGBoost regression model, post-hoc SHAP (SHapley Additive exPlanations) analysis was performed.

**Figure 9:**
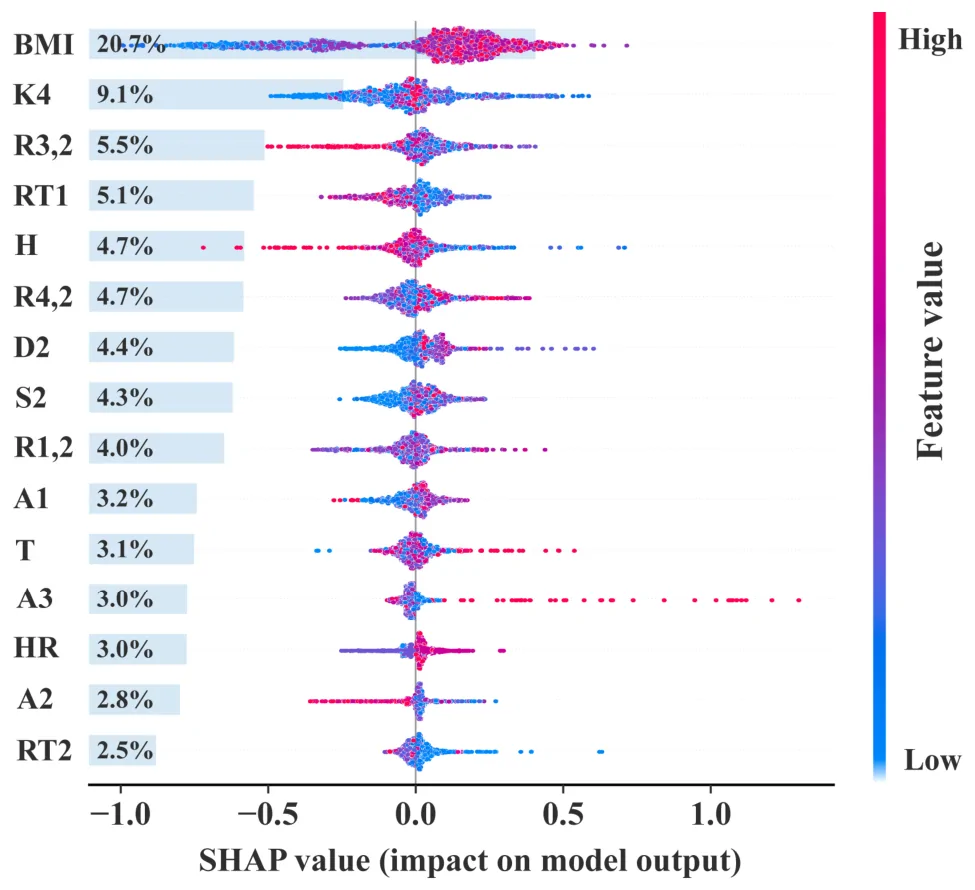
Global feature importance based on SHAP analysis.

In the SHAP-based global feature importance analysis, body mass index (BMI) ranked first, accounting for 20.7% of the total feature importance (Figure 9). Channel indices 1–4 correspond to wavelengths of 940, 1300, 1550, and 1650 nm, respectively. Among the optical features, signal kurtosis of the 1650 nm channel (K4) ranked second with an importance of 9.1%. The amplitude ratio of the 1550 nm channel to the 1300 nm reference channel (R3, 2), the amplitude ratio of the 1650 nm channel to the 1300 nm reference channel (R4, 2), and the amplitude ratio of the 940 nm channel to the 1300 nm reference channel (R1, 2) contributed 5.5%, 4.7%, and 4.0%, respectively. Pulse-wave temporal and morphological features also contributed to the model predictions. The rise time of the 940 nm channel (RT1) and the rise time of the 1300 nm channel (RT2) accounted for 5.1% and 2.5%, respectively. The mean valley value of the 1300 nm channel (D2) and the mean peak value of the 1300 nm channel (S2) contributed 4.4% and 4.3%, respectively. The average area under the rising segment at 940 nm (A1), at 1550 nm (A3), and at 1300 nm (A2) accounted for 3.2%, 3.0%, and 2.8%, respectively, while heart rate (HR) contributed 3.0%. Contact-site temperature (T) and the relative humidity of the adjacent air within the finger clip (H) accounted for 3.1% and 4.7% of the normalized global SHAP importance, respectively.

As depicted in the SHAP beeswarm distribution (Figure 9), higher BMI values (indicated by red dots) systematically exerted a positive impact on the model output, while features such as K4 exhibited distinct bidirectional SHAP value spreads, reflecting multi-factor non-linear optical interaction.

### 3.4. Cross-Session Model Stability

To evaluate the predictive performance of the proposed framework across independent acquisition sessions, a session-holdout test was conducted using acquisition groups excluded from model training and hyperparameter optimization. All preprocessing transformations were fitted exclusively on the training data and subsequently applied to the held-out test set without further adjustment, ensuring separation between model development and evaluation.

**Figure 10:**
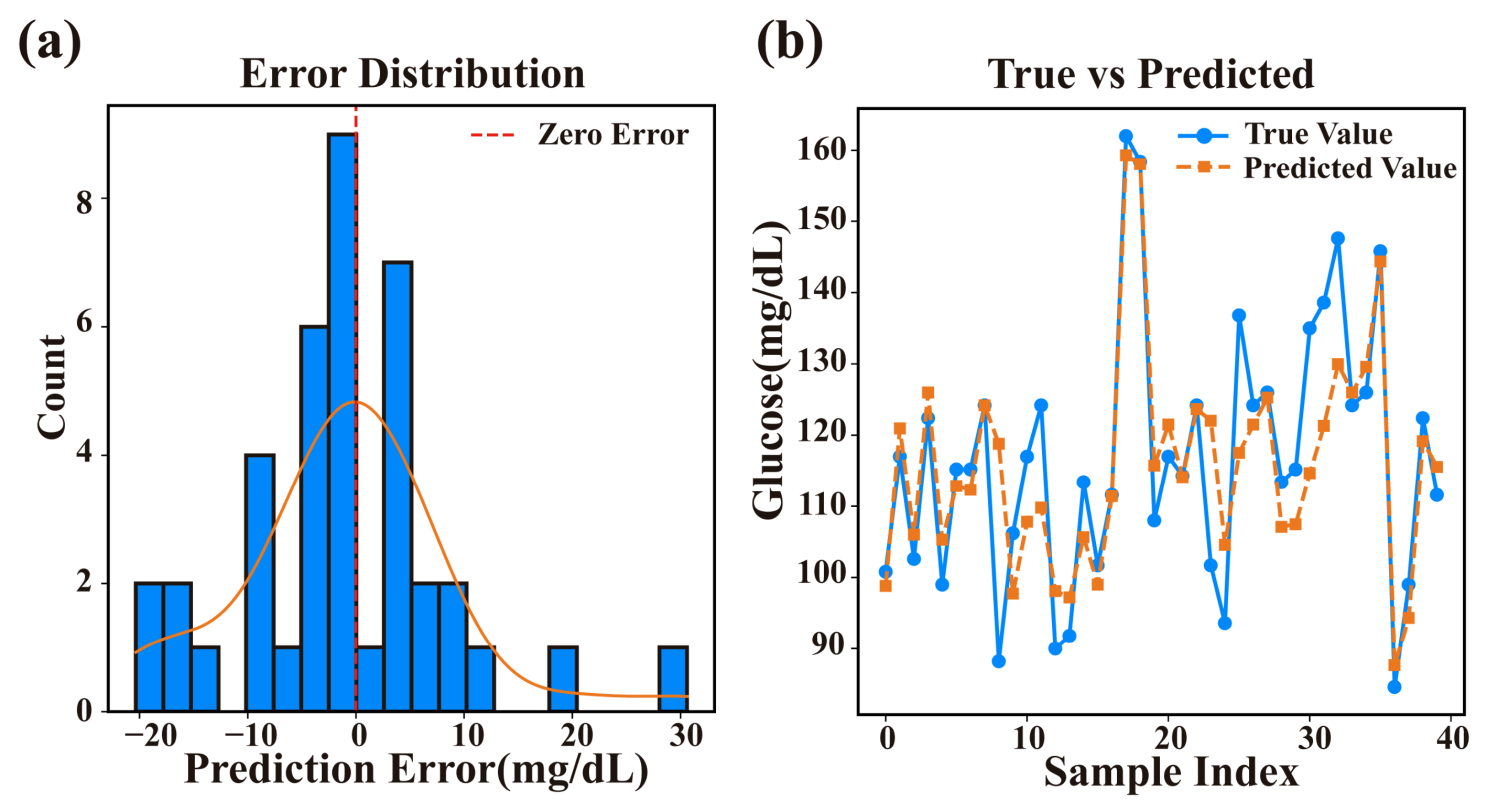
Session-holdout evaluation of the proposed model. (**a**) Distribution of prediction residuals; (**b**) comparison of predicted and reference glucose values across test sample indices.

The model was evaluated on 40 held-out samples, achieving R^2^ = 0.7261, RMSE = 0.538, and MARD = 6.07%, indicating that predictive performance was retained across the evaluated sessions. As shown in the residual distribution histogram (Figure 10a), prediction errors were approximately centered around zero and exhibited a relatively symmetric distribution, suggesting no pronounced overall systematic bias in the test set. The residuals were relatively concentrated at lower glucose concentrations, whereas greater variability was observed at higher concentrations. Furthermore, the comparison of predicted and reference glucose values across sample indices (Figure 10b) showed that the model generally followed the observed glucose variations, supporting its ability to maintain predictive performance across held-out acquisition sessions from the studied participants.

## 4. Discussion

### 4.1. Multispectral Fusion and Baseline Performance Advantages

The benchmark evaluations in Section 3.1 demonstrated the definitive superiority of the optimized XGBoost architecture (R^2^ = 0.9135, MARD = 2.71%) over traditional linear and standard regression baselines. This performance gain stems directly from the multispectral feature fusion framework across the 940, 1300, 1550, and 1650 nm bands. Glucose-induced optical attenuation in skin tissue is inherently subtle and heavily masked by background water and hemoglobin absorption. While single-wavelength or narrow-band methods suffer from severe non-linear spectral overlap, combining combination-band and overtone-band wavelengths allows the non-linear decision trees in XGBoost to capture complex cross-spectral interactions.

From a clinical perspective, 97% of predictions fell within CEGA zone A, and all predictions fell within zones A and B combined. These findings provide preliminary evidence of clinically acceptable prediction errors within the evaluated dataset. However, they should be interpreted in the context of the small study population and the limited glucose range represented. The absence of predictions in zones C–E does not establish clinical safety across broader populations or glucose ranges, particularly under hypoglycemic and markedly hyperglycemic conditions. Further validation in larger and more diverse populations, including patients with diabetes, is required to determine the generalizability and potential clinical utility of the proposed framework.

### 4.2. Environmental Feature Contributions and Possible Interface Effects

The different contributions of T and H in Section 3.2 may reflect the kinds of variation that these measurements capture alongside the PPG features. Temperature at the finger–sensor contact site can be related to peripheral blood flow and the resulting pulse waveform. Because the model already receives multispectral PPG features describing pulse amplitude, shape, and timing, some temperature-related variation may be represented in those optical measurements. This could help explain the smaller additional improvement observed when T was introduced.

Humidity, in contrast, was measured in the air immediately adjacent to the finger within the clip. Variation in this confined space may reflect changes in local moisture conditions during acquisition. Such changes could affect the optical measurement interface, including the consistency of light transmission between the finger and sensor. Their effects may not be fully described by the extracted PPG features, allowing H to provide more complementary information than T. This interpretation is consistent with the larger improvement associated with H, both when it was added to PPG-only and when it was added to PPG + T.

These results support the inclusion of local humidity as a useful predictor in the present dataset, but they do not identify the physical cause of its contribution. In particular, the sensor measured air relative humidity, not skin hydration, and optical coupling was not measured independently. The small additional gain from T in the PPG + H configuration also does not establish a synergistic interaction between the two environmental features.

### 4.3. Biophotonic Considerations and SHAP-Derived Interpretation

A central challenge in noninvasive optical glucose prediction is distinguishing glucose-related optical variations from those arising from tissue properties, vascular dynamics, and measurement conditions. SHAP analysis (Figure 9) provides insight into how the full 39-dimensional XGBoost model integrates features reflecting these different sources of variation. The resulting feature attributions quantify their contributions to model predictions and offer a basis for exploring their potential biophotonic significance.

BMI had the largest individual attribution, accounting for 20.7% of the normalized global SHAP importance. As indicated by the modified Beer–Lambert framework in Equation (1), optical measurements depend partly on the effective light path through tissue. BMI may capture participant-level differences associated with tissue characteristics that affect this path and, consequently, the interpretation of PPG signals. However, BMI does not directly measure optical path length, and its attribution may also reflect other differences between participants, including their glucose distributions. The result therefore indicates that participant-level information contributes to the full model, without establishing a specific optical calibration role for BMI.

The model also attributed predictions to features derived from the acquired multispectral PPG signals. K4 characterizes waveform kurtosis at 1650 nm, while the amplitude ratios referenced to 1300 nm describe relationships among spectral channels. Rise time and other morphological features capture further properties of the pulse waveform. These attributions are consistent with the use of complementary spectral and waveform information in the proposed fusion framework. Moreover, the separate PPG-only analysis in Section 3.2 achieved R^2^ = 0.8469 and MARD = 3.66% without BMI, other participant characteristics, or environmental inputs. This result shows that the multispectral PPG features contained predictive information independently of BMI in the evaluated dataset, although it does not measure BMI’s incremental effect within the full model.

Finally, humidity and temperature contributed to predictions made by the full model, complementing the environmental feature comparisons discussed in Section 4.2. Taken together, the SHAP and ablation analyses show that the framework uses information from optical measurements, participant characteristics, and local measurement conditions. The observed feature contributions support the rationale for multi-feature fusion; their precise physiological and optical mechanisms require direct experimental investigation.

### 4.4. Cross-Session Model Stability and Clinical Translation Considerations

A major challenge in the practical application of noninvasive optical glucose monitoring is maintaining predictive stability across measurement sessions, particularly under variations in sensor positioning, skin contact, and physiological conditions. The evaluation in Section 3.4 showed that the model retained predictive capability on held-out acquisition sessions from the studied participants, achieving R^2^ = 0.7261, RMSE = 0.538, and MARD = 6.07%. The approximately zero-centered residual distribution (Figure 10a) suggests the absence of a pronounced systematic prediction bias in the evaluated samples, while the agreement between predicted and reference glucose variations (Figure 10b) indicates that the model can capture glucose-related changes across these sessions. However, these findings do not establish generalizability to previously unseen participants or long-term stability under routine monitoring conditions.

The observed cross-session performance suggests the potential of multispectral feature fusion for improving measurement robustness. Incorporating BMI may help account for inter-individual differences in tissue optical properties and effective optical path length, while cross-wavelength amplitude ratios may reduce common-mode variations associated with optical coupling and signal intensity. Nevertheless, the extent to which these mechanisms mitigate sensor repositioning effects requires further experimental validation. Larger, more diverse cohorts and prospective subject-independent evaluations are therefore necessary to assess the framework’s robustness and potential clinical applicability.

## 5. Conclusions

This study developed a noninvasive glucose prediction framework integrating four-wavelength PPG sensing (940, 1300, 1550, and 1650 nm), multi-feature fusion, and local temperature and humidity measurements. Using the complete 39-dimensional feature set, the optimized XGBoost model achieved R^2^ = 0.9135, RMSE = 0.2991, and MARD = 2.71% in group-isolated 10-fold cross-validation. CEGA showed that 97% of predictions fell within zone A and 100% within zones A and B. A separate session-holdout evaluation yielded R^2^ = 0.7261 and MARD = 6.07%, demonstrating predictive performance on held-out acquisition sessions within the studied cohort. Temperature and humidity feature ablation, conducted without participant characteristics, showed improved prediction performance following the incorporation of local environmental measurements, with humidity yielding a greater improvement than temperature. SHAP analysis further characterized the contributions of optical, environmental, and participant-level features to the model predictions. Collectively, these findings support the potential of combining multispectral PPG sensing with physiological and local environmental information for noninvasive glucose prediction.

Nevertheless, the small, predominantly healthy cohort and limited glucose range restrict the generalizability of the findings. Moreover, the current validation strategies do not establish generalizability to previously unseen individuals or demonstrate clinical readiness. Future studies should involve larger, more diverse cohorts with broader glucose distributions and incorporate subject-independent validation and prospective longitudinal measurements to assess model generalization and temporal stability. Further investigation of skin moisture and sensor–finger coupling may also help clarify the mechanisms underlying the observed humidity-related improvements and guide sensor optimization.

## Figures and Tables

**Table 1:** Summary of participant characteristics (*n* = 10).

Parameter	Mean ± SD	Minimum	Maximum
Age (years)	24.1 ± 0.9	23	26
Height (m)	1.693 ± 0.056	1.60	1.78
Weight (kg)	63.6 ± 9.5	47.5	79
BMI (kg/m^2^)	22.55 ± 2.67	17.45	26.37

**Table 2:** Comparison of machine-learning regression models under 10-fold cross-validation.

Model	R^2^	RMSE	MAE	MSE	MARD
GRNN	0.8695	0.3657	0.2177	0.1374	3.85%
SVR	0.8710	0.3643	0.2354	0.1362	3.71%
RF	0.8846	0.3461	0.2099	0.1207	3.27%
LightGBM	0.9122	0.3015	0.1904	0.0913	2.96%
XGBoost	0.9135	0.2991	0.1740	0.0900	2.71%
BP	0.8897	0.3357	0.2004	0.1150	3.14%

## Data Availability

The data presented in this study are available on request from the corresponding author. The data are not publicly available due to privacy and ethical restrictions.
